# Intraoral occlusal adjustment time and volume required for CAD/CAM crowns fabricated with different virtual mounting methods (A randomized crossover trial)

**DOI:** 10.1038/s41405-023-00146-8

**Published:** 2023-05-10

**Authors:** Aly Ayman Mohamed Elkady, Shereen Adel Ameen, Rasha Nabil Sami

**Affiliations:** 1 PhD Candidate, Fixed Prosthodontics Department, Cairo University, Cairo, Egypt; 2 Fixed Prosthodontics Department and Vice Dean for Community Service and Environmental Development, Faculty of Dentistry, Cairo University, Cairo, Egypt; 3 Fixed Prosthodontics Department, Faculty of Dentistry, Cairo University, Cairo, Egypt

**Keywords:** Fixed prosthodontics, Fixed prosthodontics, Occlusion

## Abstract

**Objective:**

To measure the required clinical time and volume of occlusal adjustment when the maxillary cast is positioned in a virtual articulator using one of three methods: digitization of a facebow-mounted mechanical articulator (group A), virtual Bonwill triangle (group B) or a 3D face scan (group F).

**Materials and methods:**

In this randomized, triple-blind, crossover trial; 11 participants were enrolled. Every participant had one molar indicated for a single crown restoration. Three crowns were designed and milled for every participant molar totaling 33 crowns. Each of the three crowns was fabricated with the participant’s casts virtually mounted utilizing a different method. An impression was taken of the crown in place before occlusal adjustment. The occlusal adjustment was then performed and timed with the three crowns in the different groups. After the occlusal adjustment, an impression of the adjusted crown was taken. The pre-adjustment and post-adjustment impressions were digitally superimposed and the volume difference was measured. The Kruskal-Wallis test was used to compare the groups.

**Results:**

Group A showed the shortest mean adjustment time (3:44.59 ± 3:39.07) followed by group F (4:30.09 ± 2:01.50) and group B (4:35.30 ± 2:32.33). The mean adjustment volume for group A was (28 ± 19.1 mm^3^) followed by group F (30.5 ± 18.8 mm^3^) and group B (40.6 ± 29.5 mm^3^). Different virtual mounting methods had no statistically significant effect on adjustment time (*P*-value = 0.538) or adjustment volume (*P*-value = 0.490).

**Conclusions:**

A simplified approach in virtual articulator mounting appears to be justified in the construction of a single full-coverage prosthesis. Added labor, time and cost of more elaborate virtual mounting methods seem to be counterproductive.

## Introduction

Occlusal adjustments of dental restorations are often necessary; they arise intraorally despite careful laboratory technique. These interferences could be attributed to inherent and technical fabrication errors as well as errors in data transfer between the oral cavity and the laboratory environment. Accuracy of individual jaw impressions, bite registration and mounting all contribute to a compounding error in occlusal morphology. Careful mounting of articulating instrumentation allows for similar jaw movement simulation to the patient and thus reduces the occlusal inaccuracy [1].

Facebows serve to record the spatial relationship of the maxillary dental arch to cranial reference points and transfer this relationship to an articulator. This is done to minimize occlusal discrepancies between the fabricated restoration and the opposing dentition [1–3].

Digital workflows in dentistry have become widespread, owing to the increased versatility and accuracy they provide. Currently, the majority of computer-aided design (CAD) software provide a virtual articulator simulation. However, no standard workflow exists for mounting the patient’s casts onto the virtual articulator.

Several methodologies have been devised to act as a virtual facebow. Several techniques have been developed to transfer this information such as electronic jaw motion tracking devices, face scanning and radiology (lateral cephalometric radiographs and CBCT).

This research aims to evaluate a clinically accessible, simple method of mounting a patient’s facebow relationship in a virtual environment for CAD/CAM (computer-aided design/computer-aided manufacturing) restoration fabrication. Since 3D face scanning technology is now available in many smartphones, it appears to be the most accessible method for virtual alignment.

According to systematic reviews assessing the effect of facebow use in prosthodontics [4, 5], there appears to be a lack of evidence that investigates the clinical effect of facebow use in fixed prosthodontics. Thus the idea for this randomized clinical trial emerged to bridge the gap in the literature with high-level evidence.

The null hypotheses adopted by this trial were:

There will be no difference in the time required for occlusal adjustment between crowns designed with digital face scan alignment, digitized facebow mounted mechanical articulator or arbitrary positioning of the casts on a virtual articulator.

There will be no difference in the volume of occlusal adjustment between crowns designed with digital face scan alignment, digitized facebow mounted mechanical articulator or arbitrary positioning of the casts on a virtual articulator.

## Materials and methods

### Trial design

Triple blind (participant, investigator and outcomes assessor) randomized controlled crossover clinical trial with a 1:1:1 allocation ratio. The order in which the participant received the intervention was randomized and concealed from the participant, investigator and outcomes assessor.

### Sample size calculation

The power analysis used the time needed for adjustment (minutes) as the primary outcome. Based upon the results of Lin et al. [6] the mean and standard deviation values for the control group were 6.1 ± 4.8 min. Based upon expert opinion, the estimated mean occlusal adjustment time was 12 min giving a mean difference of 5.9 min from the control group. Using an alpha (α) level of (5%) and Beta (β) level of (20%) i.e., power = 80%; the minimum estimated sample size was 11 restorations per group giving a total of 33 restorations.

### Inclusion criteria

- Aged 21–70 years old, be able to read and sign the informed consent document.
- Participants with molars indicated for full coverage restorations (compromised structural integrity):
- No active periodontal or pulpal diseases
- Participants who have not undergone restorative or orthodontic treatment in the past 6 months.
- Crown to root ratio less than or equal to 1:1.
- Unrestored opposing dentition in the area of interest.
- Absence of signs of temporomandibular disorders.

### Exclusion criteria

Patients with occlusal schemes that do not permit posterior disocclusion:

- Anterior open bite.
- Edge-to-edge incisal relationship.
- Overjet > 4 mm.
- Angle’s class 2 division 1.
- Severely worn dentition.
- Class 3 occlusion with all lower anterior teeth outside of the upper anterior teeth.

### Participant recruitment

**Fig. 1:**
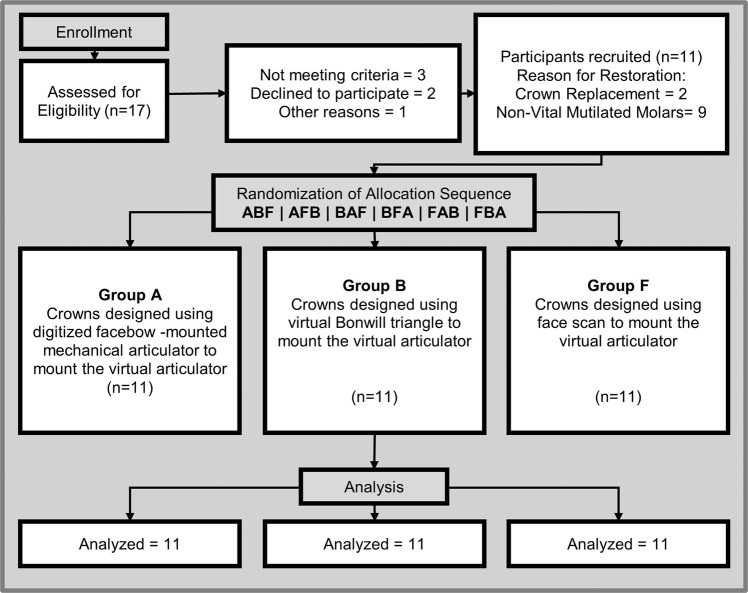
Modified CONSORT flowchart. Diagram of the progress through the phases of the crossover randomised trial of the thee groups groups (enrolment, intervention allocation, and data analysis).

This trial was conducted at the outpatient clinic of the faculty of dentistry, Cairo University between March 2021 and December 2021. Eleven participants, 2 male and 9 female (mean age 39.7 years) were enrolled. The participants met the inclusion criteria and were scheduled for single crown molar restorations. All participants were given an informed consent form approved by the faculty’s ethics committee (approval number 19-9-14). This trial required fabricating three monolithic zirconia ceramic (Katana Super Translucent Multilayer (STML) - Kuraray Noritake Dental Inc., Tokyo, Japan) crowns for each tooth. The crowns were divided into three groups, each having 11 crowns Fig. 1. Each group was designed using a different method of virtual mounting:

- **Group A**: using a digitized facebow mounted mechanical articulator.
- **Group B:** using the CAD software’s Bonwill triangle.
- **Group F:** using the participant’s face scan.

### First clinical visit

History taking, clinical examination, radiography and single-step monophase polyether primary impressions (3 M Monophase – 3 M ESPE, USA) were performed. A mechanical facebow (A7 Plus – Bioart, Brazil) registration was performed according to the manufacturer’s instructions.

**Fig. 2:**
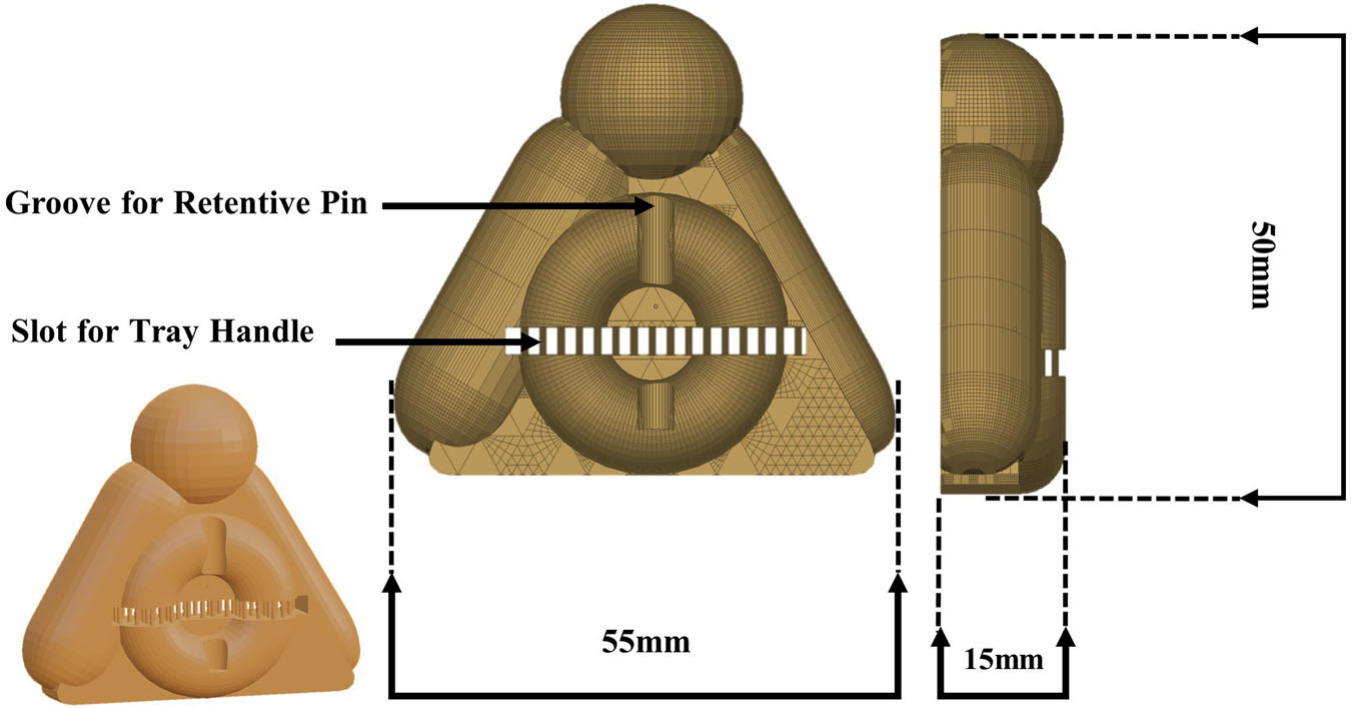
Scan marker design. Schematic diagram of scan marker design.

**Fig. 3:**
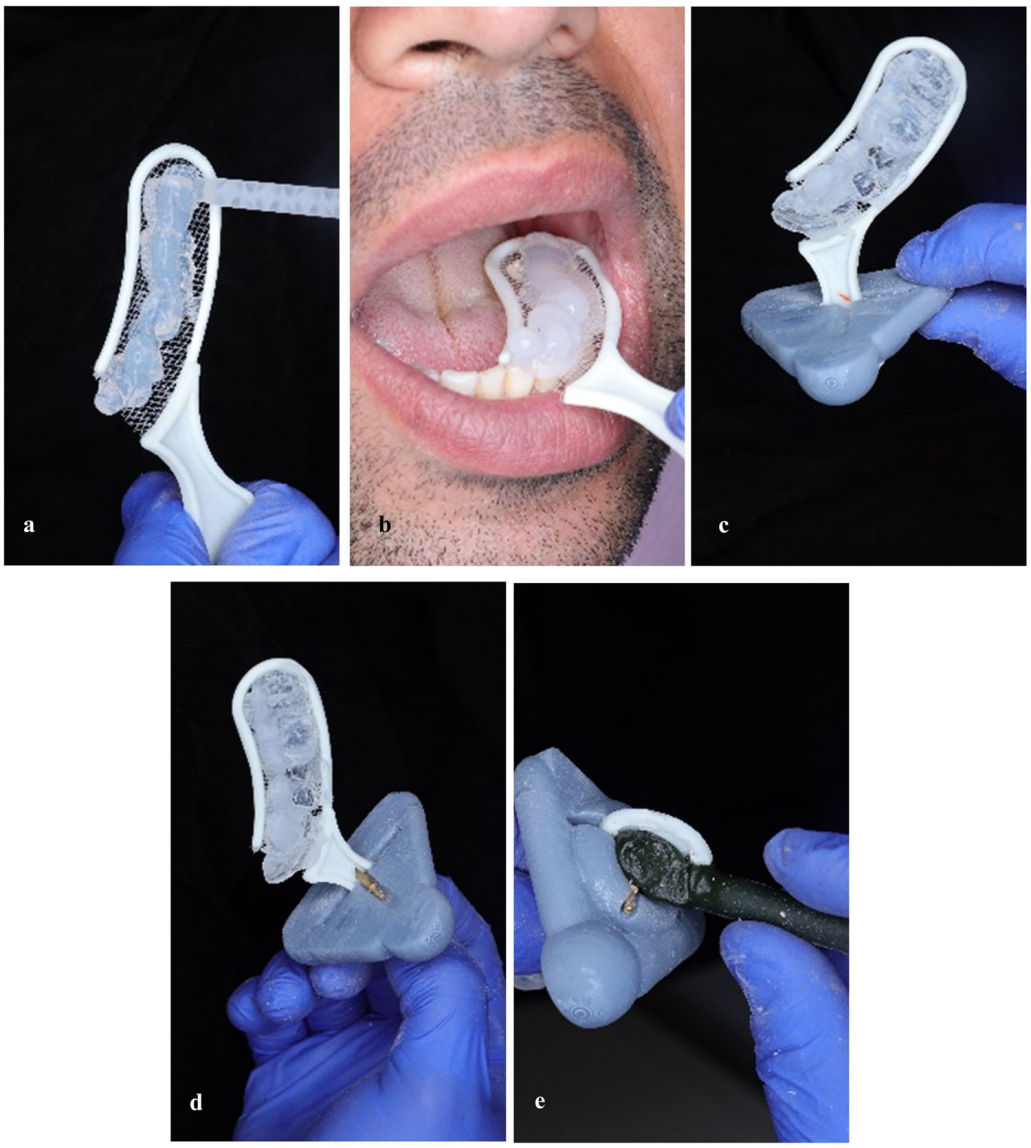
Transoral face scan appliance assembly. **a** Dispensing bite registration material. **b** Bite registration. **c** Handle of bite tray passed through slot of scan marker and hole positions marked. **d** Brass pins placed through drilled holes in the bite tray handle. **e** Stabilization of assembly with thermoplastic impression compound.

A scan marker was designed and 3D printed to fabricate a transoral appliance that will be utilized during face scanning and virtual mounting of group F Fig. 2. A bite tray (BT03 Bite Tray – Cotisen, China) was loaded and positioned to cover all the occlusal surfaces of the teeth on the left side of the mouth Fig. 3a, b. Trimming of the excess bite registration material was then performed sparing indentations of cusp tips and incisal edges.

Assembly of the transoral appliance was performed by placing the bite registration tray’s handle through the scan marker slot. Retention holes were drilled Fig. 3c and the marker was secured to the bite tray using dowel pins and impression compound (Impression Compound (Green Stick) – Kerr, Switzerland) Fig. 3d, e.

Before face scanning, the participants were instructed to wipe off all makeup and remove glasses and piercings. Participants with long hair were instructed to use hair bands to tie their hair back and veiled participants were instructed to pull back their veil to expose their ears. The participants were trained to maintain an upright seated position, with the head oriented so that their maxillary occlusal plane was parallel to the floor. The infraorbital rim of the right eye was palpated and a sticker was placed to mark the orbitale point.

A cheek retractor and the assembled transoral appliance were placed in the participant’s mouth. A smartphone (iPhone XS Max (iOS version 14.4) – Apple, USA) with a 3D scan application (Hege 3D scanner (version 1.5) - Apple App Store, developer: Marek Simonik) was used to capture the face scan. Maximum scan precision and range settings were selected and a standardized protocol was implemented during face scanning. The 3D models were exported as Stanford (.ply) files to the design workstation, labeled and stored in an encrypted folder. An allocation table was created in spreadsheet software (Excel – Microsoft, USA) where patient data was stored for future blinding and random number generation during fabrication and delivery.

Primary impressions were poured using type 4 die stone (GC FUJIROCK EP - GC, Japan). The primary maxillary cast was then mounted on the articulator using the facebow record.

### Second clinical visit

A silicone putty preparation index was used during tooth preparation to standardize reduction parameters. A secondary impression was taken, utilizing the same material and technique described for primary impressions.

A provisional crown was constructed and occlusal prematurities and interferences were removed ensuring positive proximal contacts, 3–5 intercuspal holding contacts and excursive disocclusion. The provisional crown was then cemented with temporary cement (Cavex Temporary Cement – Cavex, Netherlands).

The secondary impression was poured using the same die stone as the primary casts. Both the primary casts and the master cast were digitized with a laboratory scanner (Medit T510 - Medit, Korea). The primary casts were then positioned in the bite marks of the transoral appliance and this assembly was scanned using an intraoral scanner (Medit i500 - Medit, Korea). The mounted articulator’s upper portion was scanned using an industrial scanner (EinscanPro 2x - Shining 3D, China).

### Scan data preparation

In the dental design software (exocad (version 3.0) - exocad, Germany), the virtual articulator module was initiated and the same articulator model was selected (Bioart A7). A reference plane was generated corresponding to the articulator’s Frankfort horizontal plane (FHP). The condylar elements of the virtual articulator were exported as standard tessellation language (STL) files along with the generated plane preserving their location and orientation. These files were imported into an open-source 3D modeling software (Blender (version 2.93) - The Blender Foundation, Netherlands) and used to model an analogue to the virtual articulator to standardize mesh orientation and trimming.

**Fig. 4:**
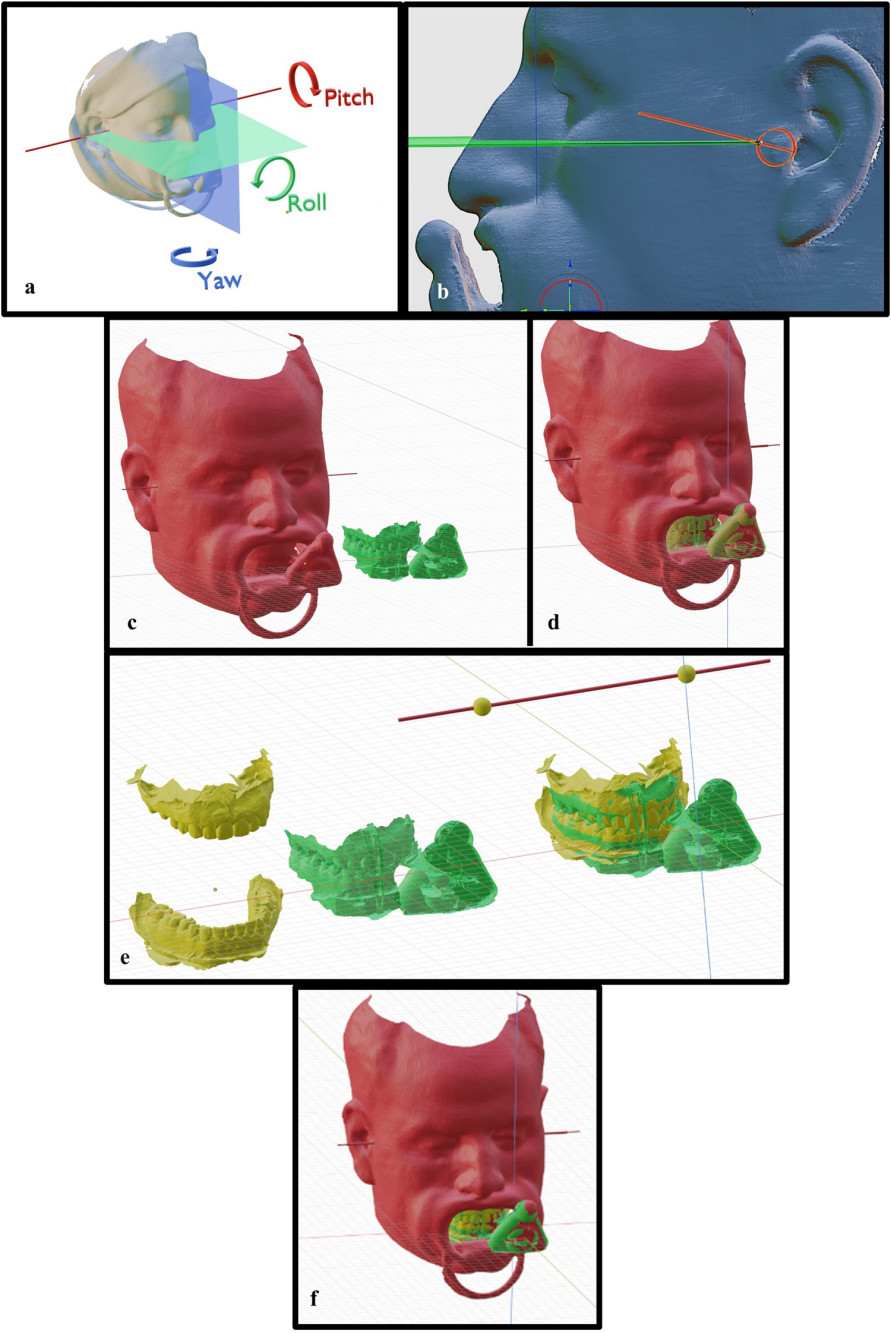
Mounting the virtual articulator using participant face scan. **a** Initial orientation. **b** Byron’s point identification. **c** Alignment of arbitrary axis and infraorbital point to articulator analogue. **d** Alignment of transoral bite scan to face scan. **e** Aligning jaw scans with transoral bite scan. **f** All scans superimposed.

In the modeling software; face scans, digitized mechanical articulator (DMA) scans and transoral appliance bite (TOB) scans were imported. The scans were trimmed to remove any unnecessary geometry. They were aligned with the virtual articulator analogue using the following steps:

1. The most superior point on the scanned external auditory meatus and the lowest point on the orbital rim of the scan were used to adjust the pitch of the face. The eyes were used to adjust the roll of the face. The facial midline was used to adjust the yaw of the face Fig. 4a.

2. An additional plane was designed to represent the canthus-tragus line onto which the determination of the arbitrary hinge axis was performed. A point 13 mm from the posterior border of the tragus was marked (Beyron’s point) on both sides of the face scan Fig. 4b.

3. A cylinder measuring 2 mm in diameter and 200 mm in length was designed and aligned to the marked hinge axis points Fig. 4c.

5. The TOB was superimposed on the face scan using the iterative closest point (ICP) alignment function between both scan marker elements Fig. 4d.

6. The face scan was hidden and the participant’s master cast scan and antagonist scan models were superimposed on the TOB scan Fig. 4e.

7. The DMA scan condylar elements were superimposed on the virtual articulator analogue.

8. The TOB, master and antagonist scans were then duplicated and aligned with the DMA scan.

9. All models were exported separately preserving their coordinates after alignment Fig. 4f.

### Computer-aided design

**Fig. 5:**
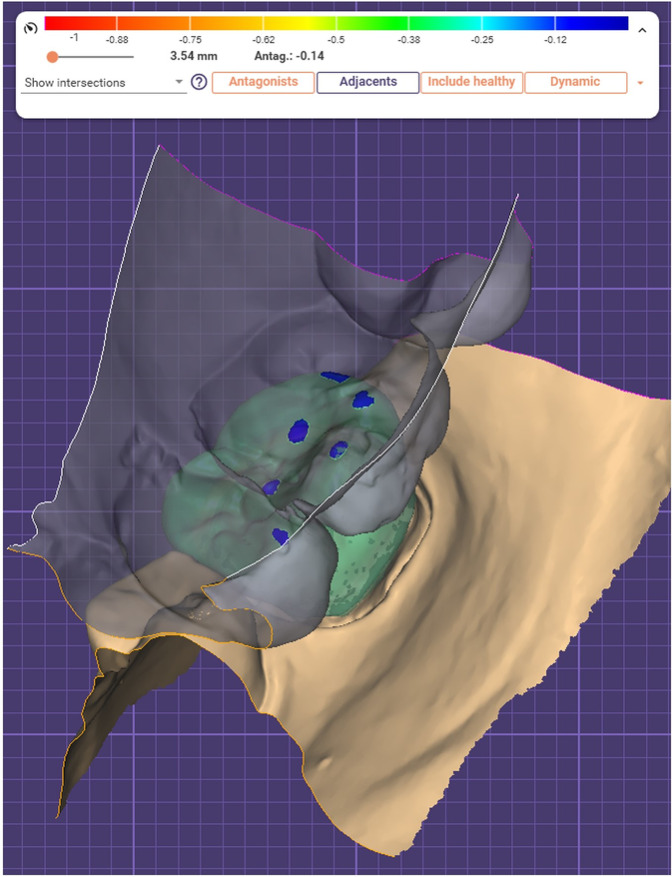
Occlusal design. Waxup design of occlusal contacts showing ~0.15 mm intersections.

A virtual wax-up was designed for each prepared molar using the dental design software. The virtual wax-up was required to demonstrate a ~ 0.15 mm intersection with its antagonist in a cusp to fossa configuration. The design had to show no other intersections with the antagonist(s) Fig. 5. The wax-up was exported to design the crowns for the different groups.

A new design project was initiated for each of the three crown designs. Average condylar path settings were programmed to be 30 ° sagittal inclination and 15 ° lateral condylar angle as recommended by Shillingburg et al. [1]. For crowns in group B (the Bonwill triangle method); the casts were aligned using the “automatic articulator alignment function” of the articulator module. This included selecting the median point between both central incisors followed by a left molar cusp tip and then a right molar cusp tip to define the occlusal plane. For crowns in group A and group F the corresponding, previously aligned master casts were imported.

**Fig. 6:**
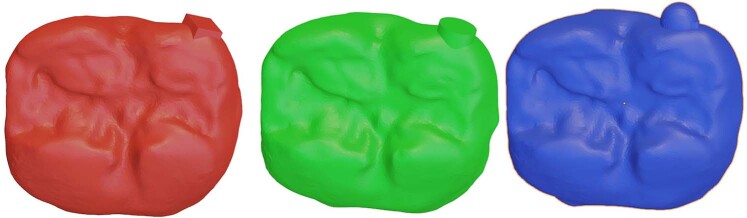
Geometric marker design. Final crown designs with appended geometric markers.

The crowns were designed by copying the wax-up. Adaptation of the design to the margin and intaglio was calculated followed by proximal and occlusal contacts. Parameters were set to 0.1 mm reduction of proximal contacts and 0 mm reduction of occlusal contacts followed by the “cut intersections” command. To ensure allocation concealment, each of the three crown designs was appended with one of three shapes; a cube, a cylinder or a sphere. The random assignment of each shape to a different group was performed for every participant. This addition was performed by the laboratory technician based on a random number sequence created on spreadsheet software by an impartial third party. This allocation was stored in a spreadsheet and concealed from the investigator/outcomes assessor. The geometric shape was placed half-buried in the distobuccal cusp of upper molars and the distolingual cusp of lower molars Fig. 6. The final design was merged and exported to an STL file for CAM processing.

All crowns were milled using the same milling machine (S1 - vhf camfacture AG, Germany). The restorations were carefully separated from the blank and support structures were removed. Only the three specimens of the same participant molar were sintered simultaneously (Tytan ZR - ROKO, Poland). The specimens were left unfinished after sintering.

### Third clinical visit

The sequence of crown delivery was randomized for every participant using a random number generated in the patient spreadsheet. The proximal contacts of the crowns were checked using articulating foil and dental floss. Restoration margins were inspected visually under 3x magnification and probed using a sharp explorer.

Once the crown was deemed clinically acceptable in regards to the aforementioned criteria, the uncemented crown was stabilized on the prepared tooth using light-consistency condensation silicone material. An impression was taken of the stabilized unadjusted crown using the same impression material and technique used for primary and secondary impressions.

The time taken to adjust occlusal prematurities and interferences was recorded using a stopwatch by the dental assistant. Only the actual intraoral occlusal adjustment time was recorded disregarding time spent drying and marking occlusal contacts. The intraoral occlusal adjustment was performed by adhering to the protocol outlined by Shillingburg [1]: The total time taken to adjust the occlusal surface of the restoration was recorded and input into the results spreadsheet.

After the occlusal adjustment was done, the quadrant was air-dried and another impression was taken including the adjusted restoration. The restoration was then removed from the set impression.

This procedure was repeated for the two remaining crowns according to the random allocation sequence. The specimen that showed the least amount of time for occlusal adjustment was selected for final cementation. The appended geometric marker on the crown was ground down and the crown’s external surface was polished. A self-adhesive resin cement (Totalcem – Itena, France) was used for the final cementation.

### Volumetric measurements

Sectional impressions of unadjusted and adjusted restorations were poured and digitized using the same desktop 3D scanner used to digitize the primary and master casts.

Each pair of quadrant casts were imported to the 3D modeling software where trimming of unnecessary mesh data was performed, leaving only the restored tooth and its mesial and distal adjacent teeth. Trimmed model files were aligned to each other using the ICP function.

**Fig. 7:**
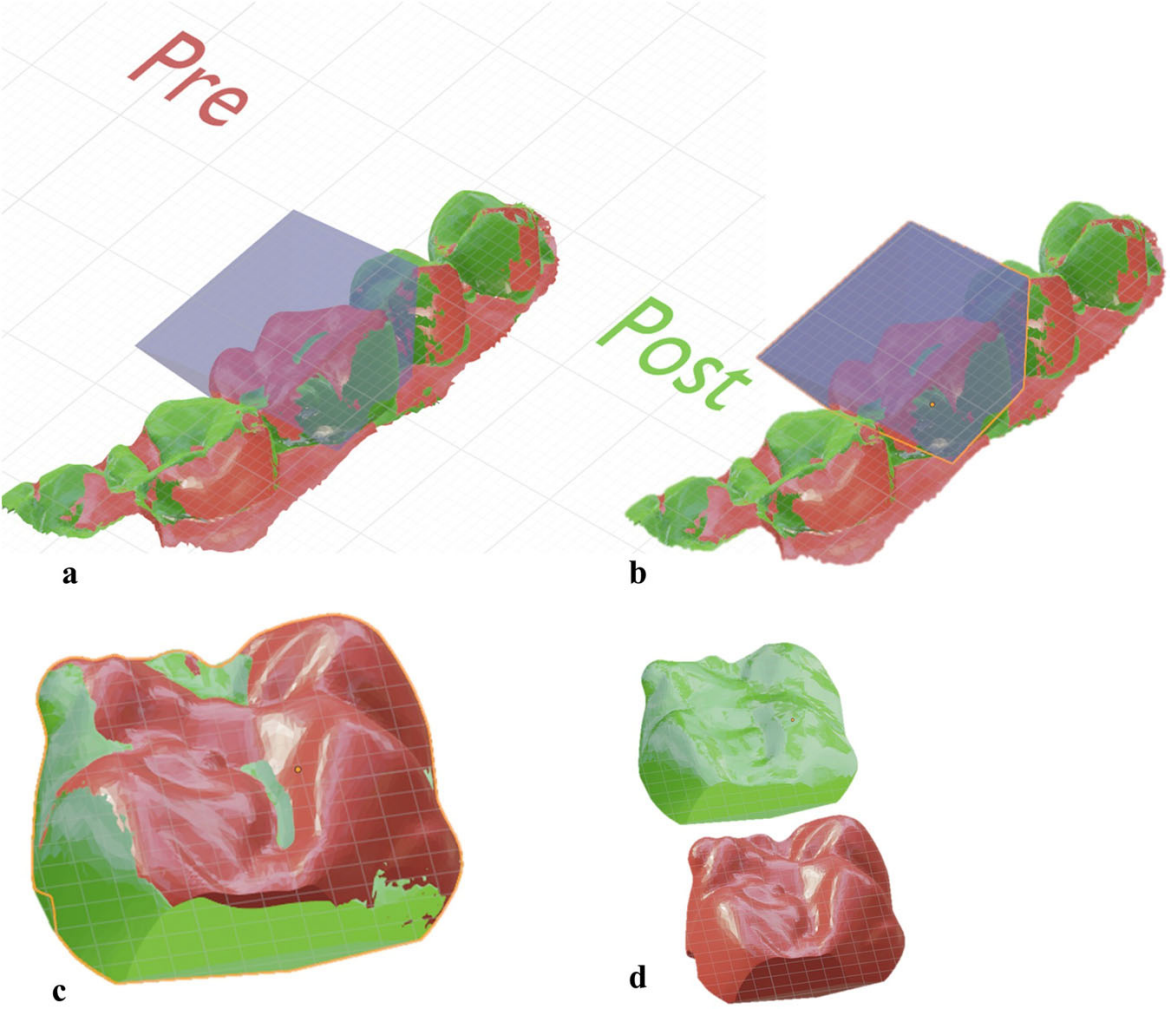
Procedural steps for determining the volumetric difference. **a** Pyramidal object designed as a cutting tool to intersect occlusal surface and functional cusp region of aligned pre-adjustment and post-adjustment scans. **b** Selection of the pyramidal cutting tool and one of the scans. **c** Isolated occlusal surfaces of both scans. **d** Scale to volume function was used to determine volume of each scan.

Quantitative volumetric analysis was performed using the same open-source 3D modeling software; a pyramidal object was generated intersecting with only the occlusal third of the restoration. The occlusal table and functional cusp slope were isolated using a Boolean intersect command. The volume of both the pre-adjustment and post-adjustment occlusal thirds was calculated using the “Scale to Volume” function. All volumetric measurements were recorded in a results spreadsheet where the difference was calculated in mm^3^ (Fig. 7a–d).

### Decoding of the results

The decoding information of which mounting method was represented by which geometric marker for each participant was released by the assisting laboratory technician. The identity of which results belong to which mounting method was input into the results spreadsheet and delivered to the statistician for analysis.

### Statistical analysis

Numerical data were explored for normality by checking the distribution of data and using tests of normality (Kolmogorov-Smirnov and Shapiro-Wilk tests). All data showed non-normal (non-parametric) distribution.

**Fig. 8:**
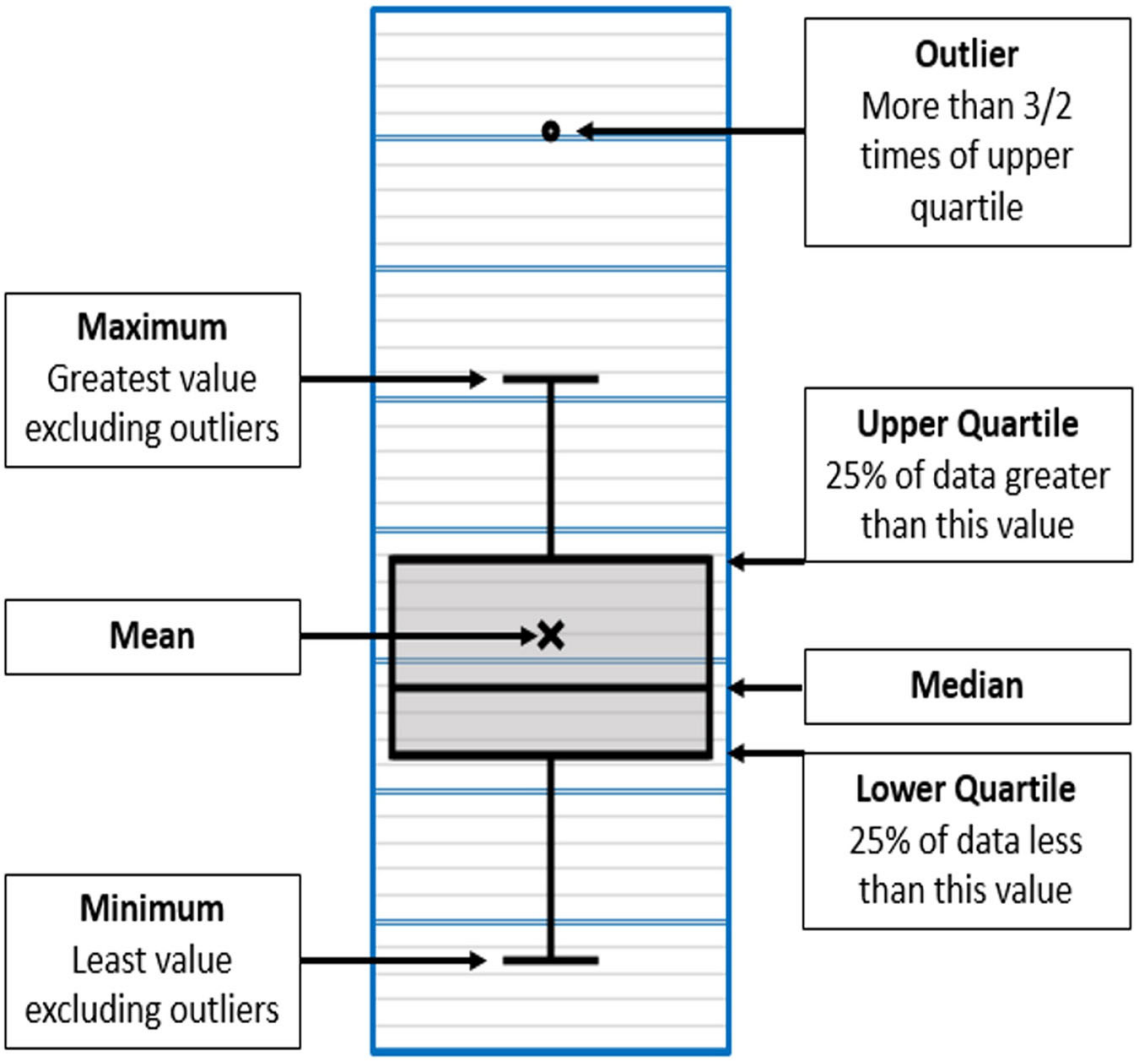
Box plot key. Diagrammatic representation of box plot interpretation.

Data were presented as mean, standard deviation (SD), median and range values then represented graphically as boxplots. In Fig. 8, a key to the interpretation of the boxplots is presented.

The Kruskal-Wallis test was used to compare the groups. The significance level was set at *P* ≤ 0.05. Statistical analysis was performed using dedicated statistical analysis software^53^.

## Results

### Occlusal adjustment time (mm:ss.ms)

**Fig. 9:**
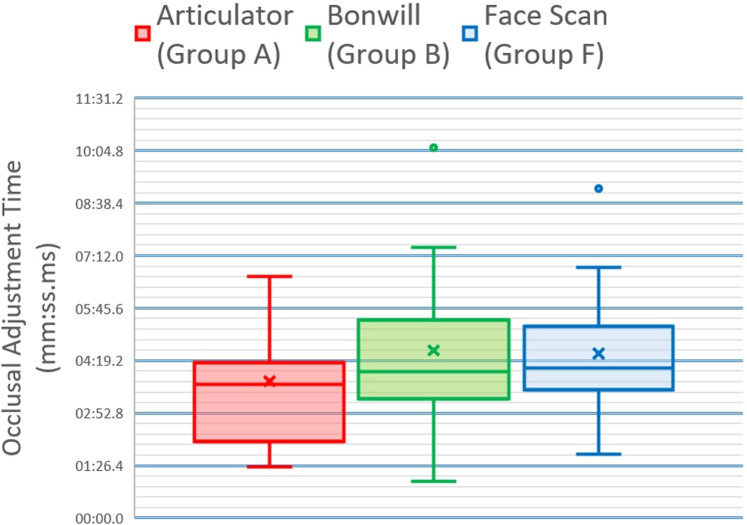
Occlusal adjustment time results. Box plot representing median and range values for occlusal adjustment time in the three groups.

Group B (virtual Bonwill triangle) showed the longest mean occlusal adjustment time (4:35.30 ± 2:32.33) whereas group A (articulator scan) showed the shortest mean occlusal adjustment time (3:44.59 ± 3:39.07) meanwhile group F (face scan) showed an intermediate mean occlusal adjustment time (4:30.09 ± 2:01.50). There was no statistically significant difference between occlusal adjustment times in the three groups (*P*-value = 0.538, Effect size = 0.025). This data is represented numerically in Table 1 and graphically in Fig. 9.

**Table 1:** Descriptive statistics and results of Kruskal-Wallis test for comparison between occlusal adjustment time (mm:ss.ms) in the three groups.

Group	Mean (mm:ss.ms)	SD (mm:ss.ms)	Median (mm:ss.ms)	Minimum (mm:ss.ms)	Maximum (mm:ss.ms)	*P*-value	Effect size (η²)
A	3:44.59	1:40.49	3:39.07	1:25.00	6:37.03	0.538	0.025
B	4:35.30	2:32.33	4:01.01	1:00.09	10:09.02		
F	4:30.09	2:01.50	4:06.09	1:44.06	9:02.09		

^*^Significant at *P* ≤ 0.05.

### Occlusal adjustment volume (mm^3^)

**Fig. 10:**
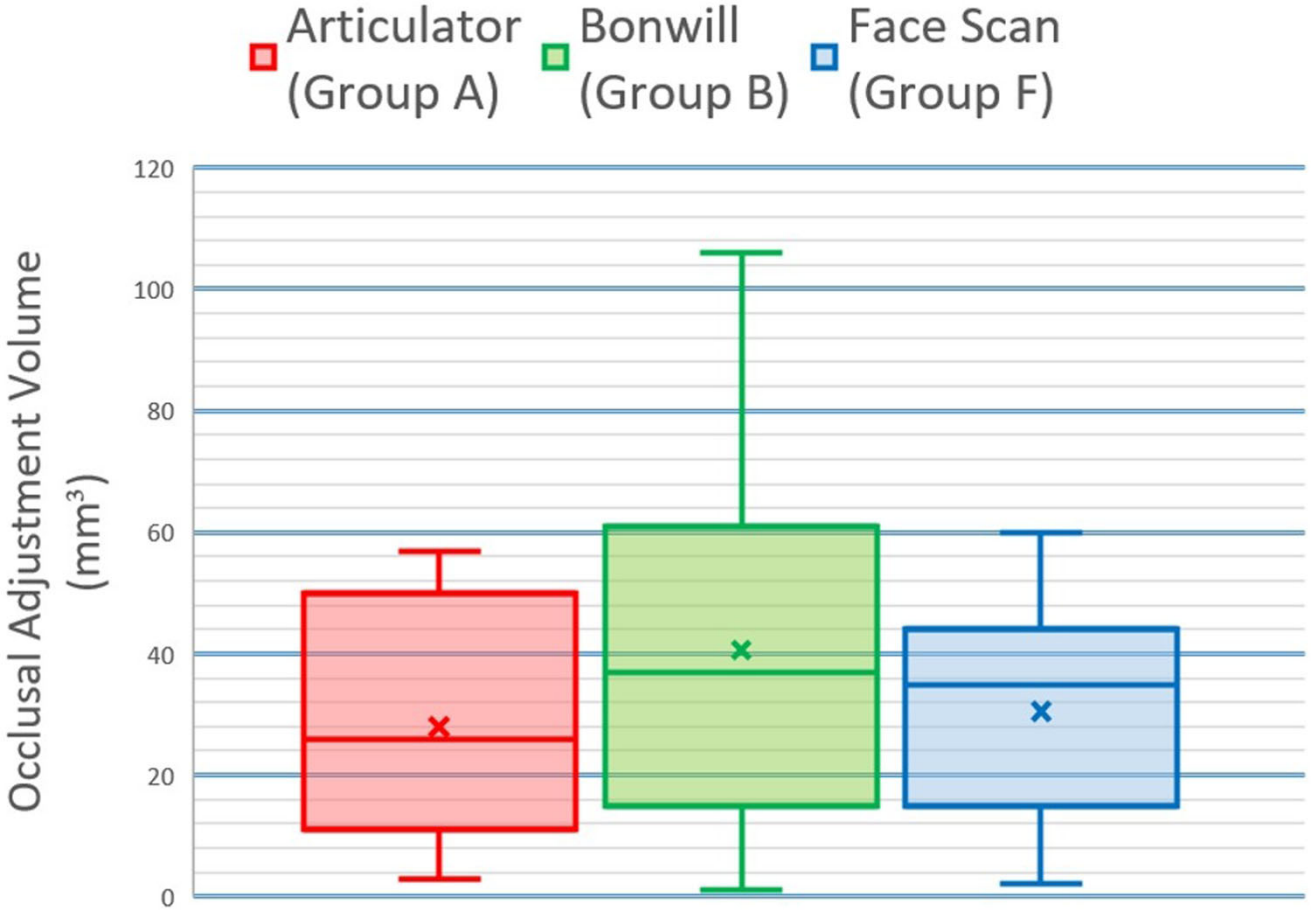
Occlusal adjustment volume results. Box plot representing median and range values for volume loss in the three groups.

Group B (virtual Bonwill triangle) showed the greatest mean occlusal adjustment volume (40.6 ± 29.5 mm^3^) whereas group A (articulator scan) showed the least mean occlusal adjustment volume (28 ± 19.1 mm^3^) meanwhile group F (face scan) showed an intermediate mean occlusal adjustment volume (30.5 ± 18.8 mm^3^). There was no statistically significant difference between volume loss in the three groups (*P*-value = 0.490, Effect size = 0.019). This data is represented numerically in (Table 2) and graphically in Fig. 10.

**Table 2:** Descriptive statistics and results of Kruskal-Wallis test for comparison between occlusal adjustment volume (mm^3^) in the three groups.

Group	Mean (mm^³^)	SD (mm^³^)	Median (mm^³^)	Minimum (mm^³^)	Maximum (mm^³^)	*P*-value	Effect size (η²)
A	28	19.1	26	3	57	0.490	0.019
B	40.6	29.5	37	1	106		
F	30.5	18.8	35	2	60		

^*^Significant at *P* ≤ 0.05.

## Discussion

It has been thoroughly documented that restorations in hyperocclusion result in tooth sensitivity, tooth hypermobility and fracture [1–3, 7]. The correlation between occlusal disharmony, cranial pain, myospasm and temporomandibular dysfunction are also topics with a long history of investigations [8–12]. The potentially harmful effects of this frequently occurring nuisance have given rise to fabrication protocols and techniques to eliminate, or at least reduce the extent of premature occlusal contacts.

Digital technology has simplified the lives of patients, clinicians and laboratory technicians alike. Still, it has yet to solve all the complex situations that are commonly presented in the dental clinic or laboratory. The transfer of this spatial relationship between the rotational axis of the jaw and the maxillary arch is the first step for the simulation of the pathways of the proposed restoration. This dictates the occlusal morphology that would not interfere during closing or lateral excursions.

Conventional mechanical mounting of casts remains the gold standard that virtual mounting has to be compared to. A 3D industrial scanner was used to digitize the facebow-mounted mechanical articulator so it can act as the comparator. Although a large field of view CBCT scan would have yielded the most anatomically-correct alignment, it was not justifiable for all participants to undergo this procedure, based on the “as low as reasonably achievable” (ALARA) principle As suggested by Colceriu-Șimon et al. [13].

Participant eligibility criteria were intended to decrease variability between the prepared teeth to their surrounding dynamic environment. The absence of opposing occlusal restorations and the posterior disclusion dynamic scheme were key factors in achieving this. The selection of only molars was important to make the occlusal area as similar as possible between participants.

Milled polycrystalline zirconia was the material of choice as its modification can affect the restoration’s mechanical and esthetic properties. the crystalline phase transformation that occurs during clinical modifications decreases the fracture toughness and ultimate strength of the restoration as advocated by Chun et al. [14] and Vila-Nova et al. [15]. Moreover, the refractive index and translucency of the restoration are also affected by the transformation of tetragonal crystals to their monoclinic form as suggested by Saker and Özcan [16].

Monolithic restorations benefit from a contracted laboratory fabrication process. Compared to pressed, cast or layered restorations; inherent material inaccuracies and human error are minimized. Confounding factors that could be introduced during fabrication by any lost wax or freehand method were eliminated to standardize restorations. No finishing, layering or glazing was performed to prevent the introduction of human elements in restoration morphology which may affect the core outcome of this study.

Face scanning with a transoral appliance (also called a scannable facebow bite fork) was utilized to provide a reference marker for the orientation of the maxillary arch as advocated by previous reports [17–23]. The design was 3D printed for standardization during scanning and subsequent alignment. The main investigator developed the face scanning strategy and the scanning strategy of the transoral bite scan using the intraoral scanner in the pilot study phase of the trial.

Although irreversible hydrocolloid primary impressions were planned, machine-mixed polyether single-step impressions were used. This decision was intended to increase the accuracy of the impression data and enhance digital superimposition results. Machine dispensing ensured standardization of proportioning and mixing of the impression material for consistent results throughout the study. The same vacuum-mixed type 4 dental stone was used for all impression pouring adhering to precise powder-water ratios. These techniques were used for all impressions performed during this study to eliminate the effects of material deformation which could affect subsequent alignment procedures.

Since the influence of the patient’s occlusion was of prime concern on the virtual articulator simulation, full arch impressions were made. A desktop scanner was therefore utilized in the digitization of the participants’ casts. This was to ensure more accurate digitization of the participants’ occlusal data. As suggested by Moon and Lee [24]; the utilization of indirect workflow in full arch digitization is more accurate than the direct workflow.

The extraoral scanner was calibrated before every scanning session to maintain a consistent level of accuracy for every scan. Using the same extraoral dental scanner was important to gain the same level of detail for all scanned models. This in turn would also aid the superimposition process.

An intraoral scanner was used to digitize the transoral bite scan because the appliance’s extraoral extension falls out of the focal region of the laboratory scanner used. This was the same reason an industrial scanner was used to scan the facebow-mounted mechanical articulator.

Digital design and computer-controlled machining were utilized with the combined digital data. For every participant, the design of each group’s specimen was nearly identical owing to the virtual wax-up copy method. The same tooth library was used for crown design to keep designs as similar as possible. This was to ensure that the automated modifications performed by the virtual articulator module were the only variable between the specimens’ design.

Milling and sintering of specimens pertaining to the same participant were done simultaneously to limit confounding variability during these processes and thus provide a comparable outcome. The same milling strategy and sintering cycle were utilized for all participants’ specimens to unify fabrication parameters.

Restorations were removed from the milling blank and only the supporting structures were removed before sintering. No surface treatment of the external surface nor intaglio was performed until all study data were collected. Utilizing the unfinished restoration would prevent any finishing procedure from altering the physical crown from the virtual design. The external surface was polished and the intaglio was air abraded and primed before resin cementation. This was to ensure a biologically compatible surface finish and greater adhesive potential of the restoration.

Clinical, laboratory and outcomes assessment procedures were performed by the same investigator to further standardize workflow parameters and decrease the effect of confounding variables that could be introduced during restoration fabrication. The only exception was the addition of the geometric coding marker on each specimen which was performed by an assisting laboratory technician. The blinding of the participant, clinician and outcomes assessor during this study was done to prevent bias during the adjustment and measurement of the specimens.

Based on the results, both null hypotheses are accepted. None of the virtual mounting methods produced superior results in terms of time or volume of occlusal adjustments of a polycrystalline zirconia CAD/CAM fabricated molar crown.

Regarding the intraoral occlusal adjustment time; on average, crowns in group A required the shortest time followed by group F and finally group B required the longest mean intraoral occlusal adjustment time. This might be explained in light of the assumption that specimens designed using the group A mounting procedure are the most anatomically similar to the participant’s articulation. This is understandable since group A represents the current gold standard mounting procedure which is based on a physical registration of anatomical landmarks. This is opposed to group B which represents the least personalized design environment for the crown specimens. The intermediate result of the crowns designed using the face scan mounting (group F) were more anatomically personalized than the arbitrary method but apparently less similar to the participant than the comparator. These results are in agreement with the traditional articulation concepts propagated in several textbooks [1–3, 25, 26]. These concepts state that utilizing a facebow produces restorations that require less occlusal adjustment. Theoretically, this is owing to the anatomical accuracy of the articulator mounting which replicates the patient’s arc of closure in the laboratory.

The lack of randomized clinical trials comparing facebow use in fixed prosthodontics has been documented in systematic reviews [4, 5]. The current body of evidence however, does include clinical trials that assess the effects of different mounting methods on the occlusal adjustments required for removable appliances. These authors were an essential inspiration for the aim and methodology of the current work. However, their results would be incomparable to this study’s results due to their heterogeneity (differences in the type of restorations and method of measuring the amount of occlusal adjustment).

The lack of statistical significance in occlusal adjustment time between groups could be attributed to the use of an earbow type of facebow; which is considered a possible source of discrepancy. Despite using a direct anatomical registration, earbows are still inherently arbitrary. This type of facebow relies on average measurements from the external auditory meatus to determine the hinge axis location. The earbow used in this study is also designed to utilize an average dimension to determine the anterior reference point which further adds to the arbitrariness of the anatomical registration. Although the use of simplified articulation instrumentation is widely advocated [1–3, 27]; some studies demonstrated a clinically and statistically significant decrease in overall occlusal adjustment by utilizing truly anatomical (kinematic) instrumentation [28]. As a consequence of this arbitrariness, some adjustment of the final crown is almost always necessary. Furthermore, there is a common belief that mandibular movements originating from centric relation are characterized by pure condylar rotation around a transverse horizontal (hinge) axis. Considering this assumption; the use of a facebow for fabricating a crown should ensure that, on the articulator, the mandibular teeth moved against the maxillary teeth on the same arc as in the patient’s mouth. However, evidence has shown that the opening-closing movement of the mandible does not occur as a pure rotation around a fixed hinge axis. A combination of condylar rotation and anterior-inferior translation of the condyle-disc complex occurs around moving instantaneous centers of rotation [29–31].

Regarding the intraoral occlusal adjustment volume, group A recorded the least mean volume followed by group F and finally group B. The volumetric results share similar justification to those outlined for the adjustment time.

Concerning the volume of occlusal adjustment; the utilization of the digital subtractive volumetric analysis method is not a novel concept. This technique has been used by Meireles et al. [32] and Kumar et al. [33] to measure erosion of tooth structure in an in vitro model. It has also been used in vivo by O’Toole et al. [34–36] where this methodology was validated against laser profilometry. Its use to measure restorative material loss during occlusal adjustment was, however, not documented to date.

The absence of statistical significance between groups may be attributed to the marked intraindividual (left/right) as well as interindividual (different participants) variability of the relationship between the condylar rotation and anterior condylar translation demonstrated during deliberate opening and closing movements as observed by Chen et al. [31]. Neither the mechanical semi-adjustable articulator nor the mathematically simulated virtual articulator used in this study can simulate these highly variable and complex opening and closing motions. Furthermore, the full arch occlusions of the participants were mounted on the articulator. This could be the reason that a facebow mounting showed no added benefit since the contralateral occlusion contributes to the constraint of articulator movement. Pesun and Swain [37] suggested that a facebow mounting would be more practical in segmental arch scan/impression situations which might provide improved constraints in the contemporary single-tooth chairside CAD/CAM workflow.

This trial has several limitations that should be acknowledged; Only single crown restorations were delivered as the intervention. The effect of mounting on the occlusal adjustment of multiunit restorations can lead to more intriguing and innovative outcomes. The use of indirect digitization instead of an intraoral scanner introduces intermediary steps, each of which has inherent inaccuracy. Eliminating the impression material shrinkage and die stone expansion would result in more accurate superimpositions and measurements. Only one type of articulator was utilized mechanically and virtually. Therefore, the results may not be generalizable to other articulators. Other techniques of virtual mounting were not investigated; using still images, radiographs, perioral scanning and jaw tracking equipment could have yielded different results. Additionally, future research could examine other factors that may impact the relationship between virtual mounting and occlusal adjustment, such as selection of different arbitrary hinge axes.

Although seemingly simple, it is undeniable that the occlusal morphology and its adjustment play a major role in the success and failure of a restored tooth. Pragmatic investigation of this vast multifactorial field seems to be lacking but necessary.

## Conclusions

Within the limitations of this study, considering the complex multistep design, the following conclusions could be drawn:

- There is no substantial difference in occlusal adjustment time when utilizing different virtual articulator mounting methods.
- The face scan method of virtual articulator mounting showed no beneficial decrease in occlusal adjustment volume nor time of digitally fabricated monolithic zirconia single crowns.
- The use of average values for arbitrary virtual articulator mounting is expected to produce single molar crowns that require similar time and volume of occlusal adjustment compared to crowns with anatomically-mounted articulators.
- It is recommended to utilize the more economical and less time-consuming arbitrary method of virtual articulator mounting (using the virtual Bonwill triangle) during single molar crown fabrication.

## Data Availability

The data that support the findings of this trial are available from the corresponding author upon reasonable request.
